# Supplementary material for: Controllable water surface to underwater transition through electrowetting in a hybrid terrestrial-aquatic microrobot
Source: Nat Commun. 2018 Jun 27;9:2495. doi: 10.1038/s41467-018-04855-9 (PMC6021446; doi:10.1038/s41467-018-04855-9)
Supplement: Supplementary file 8 — Supplementary Information [file 41467_2018_4855_MOESM8_ESM.docx]

**Supplementary Information**

**Controllable water surface to underwater transition through electrowetting in a hybrid terrestrial-aquatic microrobot**

**Chen et al.**

Supplementary Information contains:
Supplementary Figures 1-4
Supplementary Tables 1-2
Supplementary Notes 1-3
Supplementary References


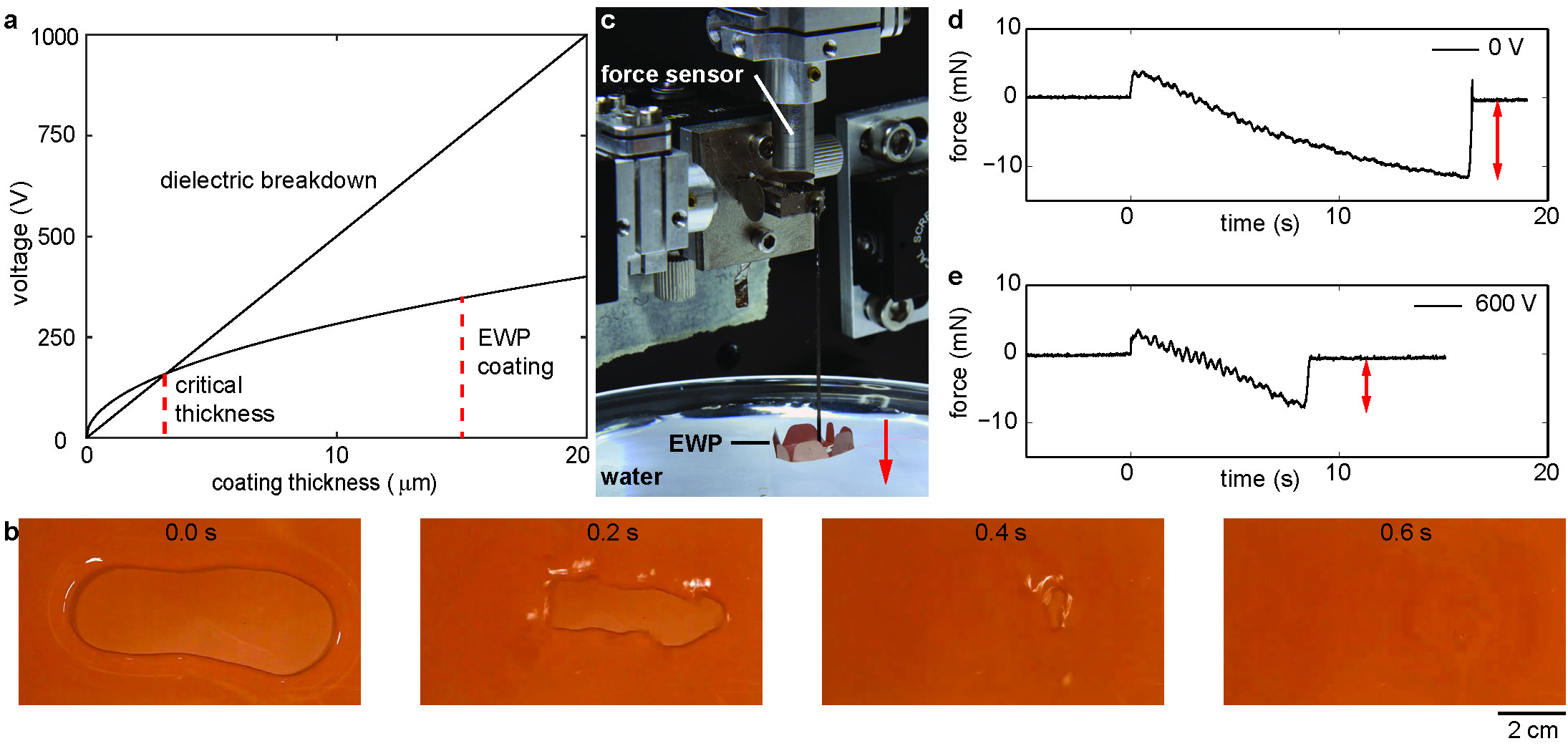


**Supplementary Figure 1: Electrowetting experiments.** (**a**) The design criteria for EWP’s Parylene coating thickness. The coating must be thicker than the critical thickness to prevent dielectric breakdown during operation. (**b**) Spontaneous wetting on a copper sheet coated by 15 μm Parylene. The surface is completely wetted within 0.6 s as a 600 V signal is sent to the copper substrate. (**c**) Experimental setup for quantifying the effects of electrowetting on an EWP. An EWP is mounted on a single axis force sensor as it is gradually pushed into water. (**d**) A maximum of 12 mN upward force is exerted by the water surface when no voltage is applied during the transition process. (**e**) The maximum upward force reduces to 8 mN when the input voltage is set to 600 V.


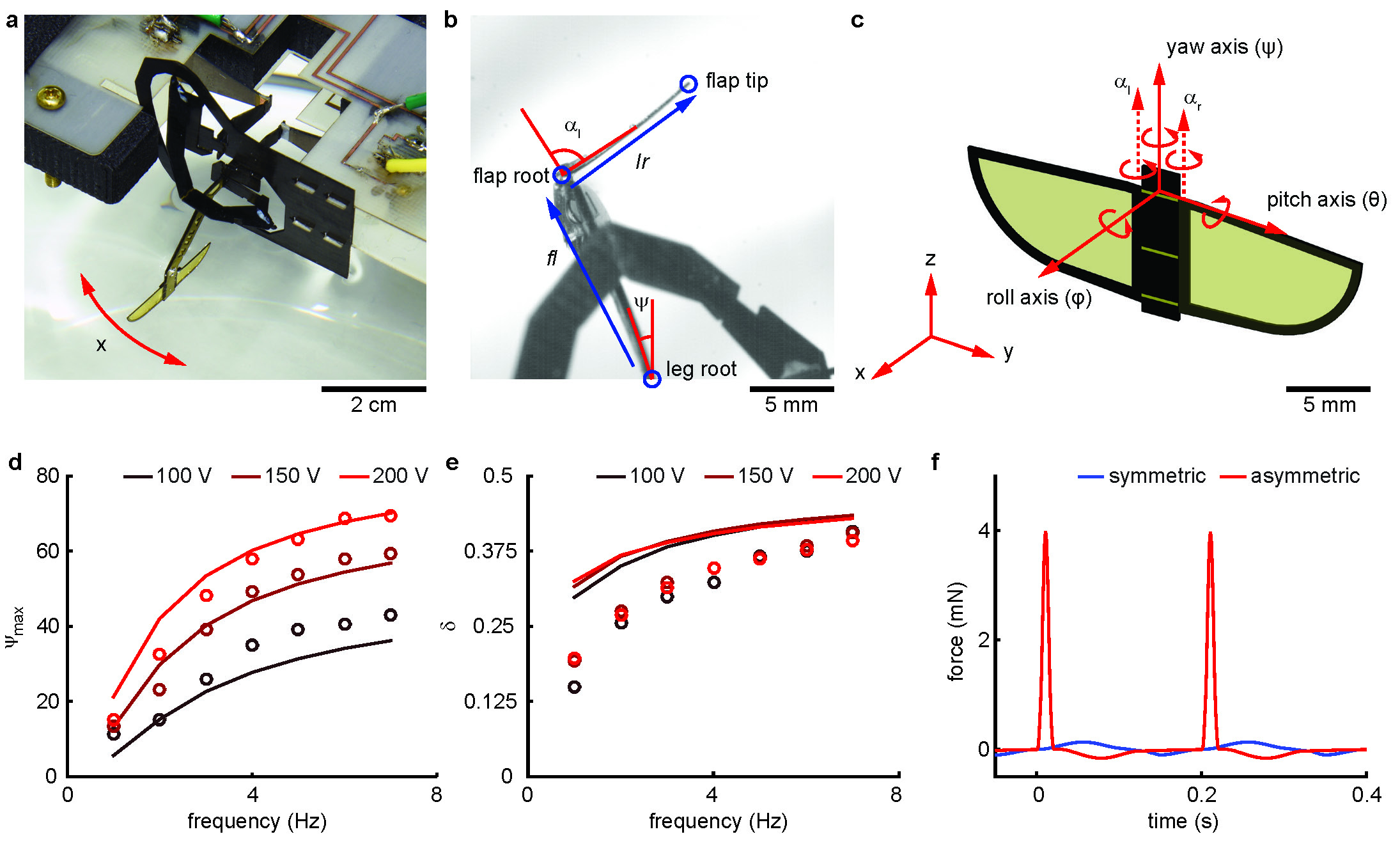


**Supplementary Figure 2: Comparison of flapping experiments and quasi-steady simulations.** (**a**) A single leg experimental setup for investigating the robot leg’s performance in water. (**b**) Annotated top view of a moving leg in water captured by a high speed camera. (**c**) The coordinate definition of the quasi-steady model. (**d, e**) Comparison between experimental and simulated maximum rotation angle of the left flap and relative phase shift between the leg motion and the flap motion, respectively. Data presented as a function of driving frequency for three different voltage amplitudes. (**f**) Comparison of simulated thrust forces between a symmetric and an asymmetric driving signal.


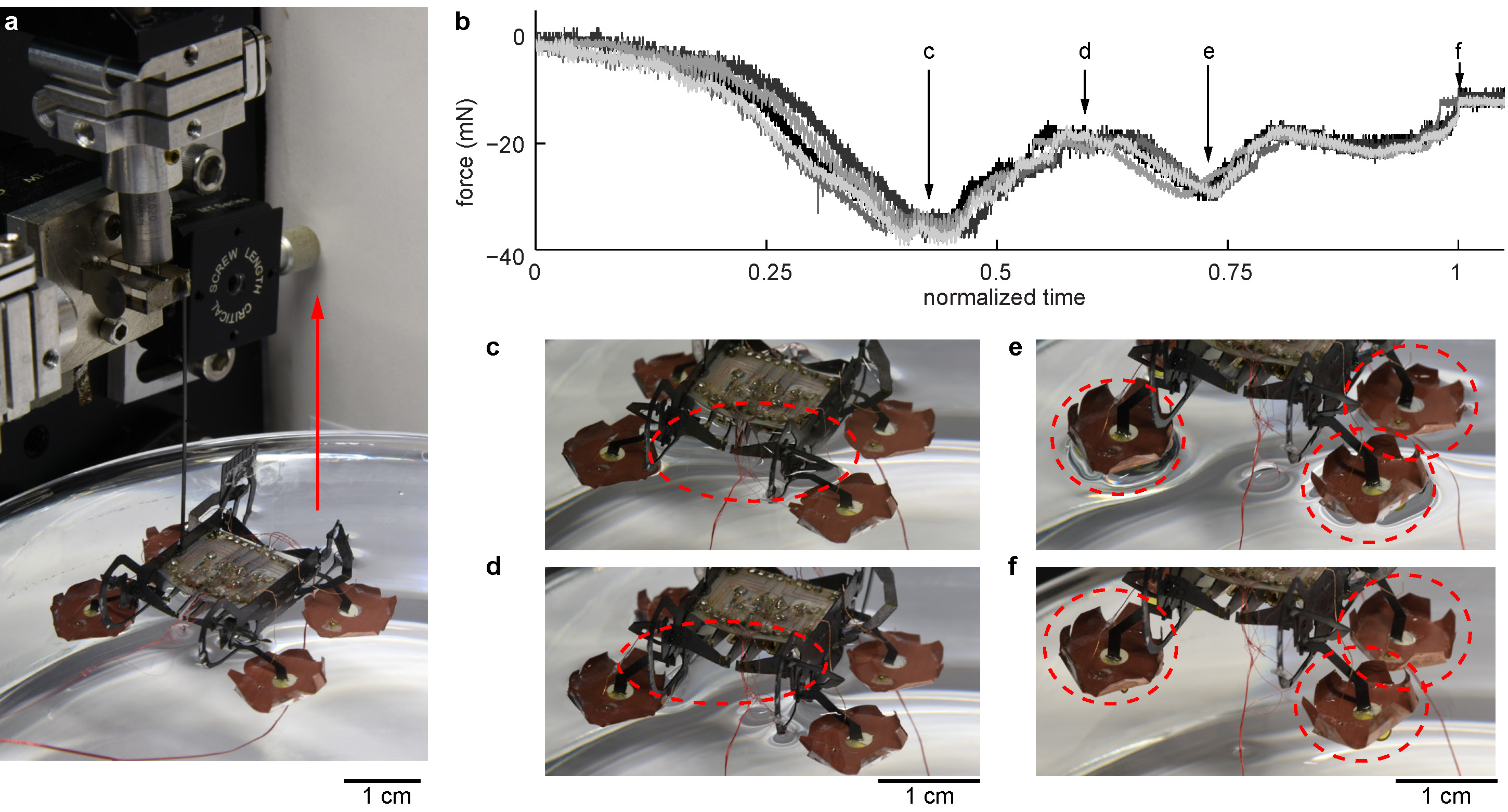


**Supplementary Figure 3: Measurement of surface tension force on a robot emerging from the water surface.** (**a**) Experimental setup for quantifying the surface tension on a robot during underwater-to-land transition. A robot is mounted on a force sensor as it is gradually pulled out of water. (**b**) Five unfiltered force measurements as the robot is pulled out of water. The events labelled by (**c-f**) correspond to the panels **c-f**. (**c-d**) The surface tension force on the robot chassis is given by the force difference between c and d. (**e-f**) The surface tension force on the robot’s EWPs is given by the force difference between **e** and **f**.


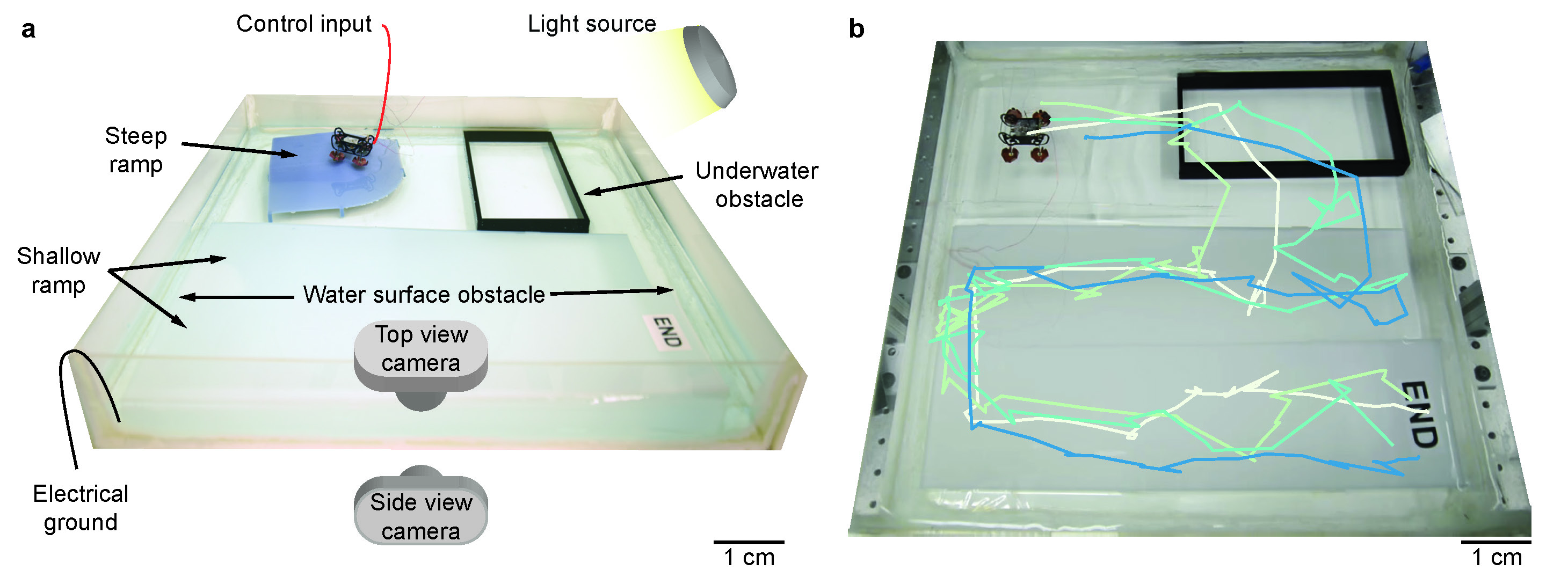


**Supplementary Figure 4: Experimental setup and repeatability of robot locomotion demonstration.** (**a**) Illustration of the experimental setup. The robot starts on a steep ramp, walks down onto the water surface, swims on the surface to evade an underwater obstacle, then sinks into the aquarium bottom, and finally walks on an incline to transition back to land. (**b**) The demonstration is repeated four times. The robot trajectories are manually tracked and overlaid on the same image.

**Supplementary Table 1.** Values of the EWP’s design parameters and physical constants

| Symbol | Parameter name | Value |
| --- | --- | --- |
| $L$ | EWP’s contact length | 45 mm |
| $A$ | EWP’s area | 150 mm^2^ |
| $d_{H}$ | Parylene coating thickness | 15 µm |
| $\gamma$ | Water surface tension coefficient | 7.29e-2 N m^-1^ |
| $\rho_{w}$ | Density of water | 1000 kg m^-3^ |
| $g$ | Gravity | 9.8 m s^-2^ |
| $\theta_{N}$ | Nominal contact angle of water on Parylene | 140° |
| $\epsilon_{0}$ | Permittivity of free space | 8.85e-12 F m^-1^ |
| $\epsilon_{l}$ | Relative permittivity of Parylene | 3.15 |

**Supplementary Table 2.** Robot cost of transport in different environments under different inputs

| Terrains | Surface | Gait | Frequency (Hz) | Speed (cm s^-1^) | Power (mW) | Cost of transport |
| --- | --- | --- | --- | --- | --- | --- |
| Ground | Acrylic | Trot | 1 | 0.7 | 1.1 | 9.6 |
| Ground | Acrylic | Trot | 6 | 4.2 | 6.2 | 9.4 |
| Ground | Acrylic | Trot | 10 | 7.0 | 9.6 | 8.8 |
| Underwater | Acrylic | Trot | 1 | 0.5 | 1.1 | 13.5 |
| Underwater | Acrylic, 3° incline | Trot | 4 | 0.9 | 5.2 | 37.0 |
| Water surface | ---------- | Swim | 5 | 2.8 | 4.8 | 10.9 |

**Supplementary Note 1: Derivation of the dynamical model**

**Notations**

We use the following notations for the derivations:

We use bold letters to denote vector quantities and hat to denote unit vectors. **0** denotes a vector of all 0s. For the special unit vector that is parallel to a particular coordinate axis, we write the vector as **e_i_** without a hat. It means the vector is 1 at the i^th^ entry and 0 everywhere else.

- We use upper case letters to represent matrix quantities. Specifically, we use *R* to denote a $3\times3$ rotation matrix.
- For vectors $\mathbf{v}\in\mathbb{R}^{3}$, $\mathbf{a}\in\mathbb{R}^{3}$, we let $S_{\mathbf{v}}$ denote the skew symmetric matrix generated by **v** such that $S_{\mathbf{v}}=\mathbf{v}\times\mathbf{a}$.

**Dynamical model of the robot leg and passive flaps**

We formulate a time varying model that has eight degrees of freedom. The robot leg has six translational and rotational degrees of freedom denoted by $x$, $y$, $z$, $\phi$, $\theta$, and $\psi$. Here $x$, $y$, $z$ are the translational degrees of freedom and $\phi$, $\theta$, $\psi$ are Euler angles following the roll, pitch, and yaw convention. Supplementary Figure 2c defines the robot leg and flaps’ rotational axes. The robot flap kinematics each have one degree of freedom relative to the robot leg for a total of two degrees of freedom corresponding to the robot's left and right flaps. Here $\alpha_{r}$ and $\alpha_{l}$ denote the right and the left flap rotational angles, respectively. These eight generalized coordinates are defined as a column vector:

$\boldsymbol{q}=\left[ x,y,z,\phi,\theta,\psi,\alpha_{r},\alpha_{l} \right]^{T}$ . (1)

**Rigid body dynamics**

We adopt the matrix form of Lagrangian mechanics to derive the equation of motion^1^:

$D\left( \boldsymbol{q} \right)\ddot{\boldsymbol{q}}+C\left( \boldsymbol{q},\dot{\boldsymbol{q}} \right)\dot{\boldsymbol{q}}+\boldsymbol{g}\left( \boldsymbol{q} \right)=\boldsymbol{\tau}$ . (2)

The inertia matrix D is given by the sum of the contributions from leg, right and left flaps:

$D= \sum_{i\in\left\{ b,r,l \right\}} (m_{i}J_{v,i}^{T}J_{v,i}+J_{w,i}^{T}R_{i}I_{i}R_{i}^{T}J_{w,i})$, (3)

where the subscripts $b, r, l$ denote the robot leg, its right and left flaps, respectively. The symbols $m_{i}$ and $I_{i}$are the mass and moment of inertia of component $i$. *R_i_* is the corresponding rotation matrix from the center of mass reference frame to the inertial system. *J_v,i_* and *J_w,i_* are velocity and angular velocity Jacobians. The Christoffel matrix elements are obtained from the partial differentials of the inertia matrix:

$C_{kj}=\sum_{i=1}^{n} C_{ijk}\left( \mathbf{q} \right)q_{i}=\sum_{i=1}^{n} \frac{1}{2}\left( \frac{\partial D_{kj}}{\partial q_{i}}+\frac{\partial D_{ki}}{\partial q_{j}}+\frac{\partial D_{ij}}{\partial q_{k}} \right)q_{i}$ (4)

The j^th^ component of the gravity vector is given by:

$g_{j}=\frac{\partial P}{\partial q_{j}}$, (5)

where *P* is the total potential energy. The generalized force vector $\mathbf{\tau}$ is given by the matrix product of the partial displacement matrix and the external force vector. The dimensionalities of these quantities are given by: $D\in\mathbb{R}^{8\times8}$, $C\in\mathbb{R}^{8\times8}$, $\boldsymbol{g}\in\mathbb{R}^{8\times1}$, and $\boldsymbol{\tau}\in\mathbb{R}^{8\times1}$. The equations of motion form a system of coupled ordinary differential equations. We solve this system numerically through the matlab function ode45.

Next, we derive the velocity Jacobians and angular velocity Jacobians of the robot leg, its right and left flaps.

**Leg** $\boldsymbol{J}_{\boldsymbol{v}}$**and** $\boldsymbol{J}_{\boldsymbol{w}}$

The robot leg velocity Jacobian *J_v,b_* transforms velocities with respect to the generalized coordinate to velocities with respect to the inertial coordinate. Here we have $J_{v,b}\in\mathbb{R}^{3\times8}$ and it is given by:

$J_{v,b}=\left( \begin{matrix} | & | & | & | & | & | & | & | \\ \boldsymbol{e}_{\boldsymbol{1}} & \boldsymbol{e}_{\boldsymbol{2}} & \boldsymbol{e}_{\boldsymbol{3}} & \boldsymbol{0} & \boldsymbol{0} & \boldsymbol{0} & \boldsymbol{0} & \boldsymbol{0} \\ | & | & | & | & | & | & | & | \end{matrix} \right)$. (6)

The body angular velocity Jacobian is given by

$J_{w,b}=\left( \begin{matrix} | & | & | & | & | & | & | & | \\ \boldsymbol{0} & \boldsymbol{0} & \boldsymbol{0} & R_{\psi}R_{\theta}\boldsymbol{e}_{\boldsymbol{1}} & R_{\psi}\boldsymbol{e}_{\boldsymbol{2}} & \boldsymbol{e}_{\boldsymbol{3}} & \boldsymbol{0} & \boldsymbol{0} \\ | & | & | & | & | & | & | & | \end{matrix} \right)$, (7)

where $R_{\psi}$ and $R_{\theta}$ are rotation matrices with respect to the $\psi$ and $\theta$ axes. In the next sections we will use the rotation matrices $R_{\phi}$, $R_{\alpha r}$, and $R_{\alpha l}$. These are rotations with respect to the axes defined in Supplementary Figure 2c.

**Robot leg and flap displacement vectors**

The right and left flap velocity and angular velocity Jacobians contain a number of displacement vectors that define the distance between the robot leg center of mass, flap root, and flap center of mass. These vectors are defined as:

- $\mathbf{d}_{rb}$: displacement from the leg center of mass to the right flap center of mass
- $\mathbf{d}_{lb}$: displacement from the leg center of mass to the left flap center of mass
- $\mathbf{d}_{rf}$: displacement from the right flap root to the right flap center of mass
- $\mathbf{d}_{lf}$: displacement from the left flap root to the left flap center of mass

These displacement vectors can be calculated from a sequence of translations and rotations from the default robot configuration. Let $\boldsymbol{r}_{rb}$, $\boldsymbol{r}_{lb}$, $\boldsymbol{r}_{rf}$, and $\boldsymbol{r}_{lf}$ be the corresponding robot parameters in the fixed frame. The transformations are given by:

$d_{rb}=R_{b}\left( r_{rb}-r_{rf} \right)+R_{b}R_{\alpha r}r_{rf}$, (8)

$d_{lb}=R_{b}\left( r_{lb}-r_{lf} \right)+R_{b}R_{f}R_{\alpha r}r_{lf}$, (9)

$d_{rf}=R_{b}R_{\alpha r}r_{rf}$, (10)

$d_{lf}=R_{b}R_{f}R_{\alpha l}r_{lf}$, (11)

where $R_{b}$ is the body rotation matrix:

$R_{b}=R_{\psi}R_{\theta}R_{\phi}$. (12)

The rotation matrix $R_{f}$ accounts for the 180° rotation between the left flap coordinate and the body coordinate.

**Jacobian matrices of the right flap**

The coordinate orientation of the right flap is the same as that of the robot leg. The flap rotates with respect to the axis *z_r_*. The right flap velocity Jacobian is given by:

$J_{v,r}=\left( \begin{matrix} | & | & | & | & | & | & | & | \\ e_{1} & e_{2} & e_{3} & R_{\psi}R_{\theta}S_{e_{1}}d_{rb} & R_{\psi}S_{e_{2}}d_{rb} & S_{e_{3}}d_{rb} & R_{b}S_{e_{3}}d_{rf} & 0 \\ | & | & | & | & | & | & | & | \end{matrix} \right).$ (13)

The right flap angular velocity Jacobian is given by:

$J_{w,r}=\left( \begin{matrix} | & | & | & | & | & | & | & | \\ 0 & 0 & 0 & R_{\psi}R_{\theta}e_{1} & R_{\psi}e_{2} & e_{3} & R_{b}e_{3} & 0 \\ | & | & | & | & | & | & | & | \end{matrix} \right).$ (14)

**Jacobian matrices of the left flap**

The left flap coordinate system is rotated 180º along the $z_{l}$axis compared to the robot leg coordinate system. The left flap rotates with respect to the axis *z_l_*. The left flap velocity Jacobian is given by:

$J_{v,l}=\left( \begin{matrix} | & | & | & | & | & | & | & | \\ e_{1} & e_{2} & e_{3} & R_{\psi}R_{\theta}S_{e_{1}}d_{lb} & R_{\psi}S_{e_{2}}d_{lb} & S_{e_{3}}d_{lb} & 0 & R_{b}{R_{f}S}_{e_{3}}d_{lf} \\ | & | & | & | & | & | & | & | \end{matrix} \right).$ (15)

The left flap angular velocity Jacobian is given by:

$J_{w,l}=\left( \begin{matrix} | & | & | & | & | & | & | & | \\ 0 & 0 & 0 & R_{\psi}R_{\theta}e_{1} & R_{\psi}e_{2} & e_{3} & 0 & R_{b}R_{cf}e_{3} \\ | & | & | & | & | & | & | & | \end{matrix} \right).$ (16)

**The generalized force** $\boldsymbol{\tau}$

To solve for the motion of the right and the left flaps, we need to find the generalized force $\boldsymbol{\tau}$ and invert the matrix system given by Supplementary Equation (2). The generalized force relates to the net external force, and it is given by the equation:

$\tau_{i}=\sum_{j=1}^{3} F_{ext,j}\frac{\partial r_{j}}{\partial q_{i}}$, (17)

where $F_{ext,j}$ is the j^th^ component of the net external force, and $\frac{\partial r_{j}}{\partial q_{i}}$ is the transformation from the inertial coordinates to the generalized coordinates.

The net external force $\boldsymbol{F}_{ext}$ consists of aerodynamic forces, flexure viscoelastic forces, and actuator driving forces:

$\boldsymbol{F}_{ext}=\boldsymbol{F}_{aero}+\boldsymbol{F}_{flexure}+\boldsymbol{F}_{act}$. (18)

Instead of modeling the actuator forces, we assume that the actuator is powerful enough to directly control the leg displacement and rotation. Previous studies have shown that the actuators are capable of delivering instantaneous forces larger than 12 mN at the robot leg. In all of our simulations, the peak force at the robot leg is limited to 4 mN. This condition implies that the assumption of having ideal, displacement based actuators is reasonable for our application. Instead of solving for all eight kinematic variables, we only solve for the passive right and left flap rotation while treating the leg motion as inputs.

This simplification still requires us to find the terms $\boldsymbol{F}_{aero}$ and $\boldsymbol{F}_{flexure}$. We describe the modeling of these external forces in the following sections.

**A quasi-steady model**

We use a blade element, quasi-steady model to calculate the net drag force on the robot leg, the right flap and the left flap. The blade element model divides the robot leg and flaps into vertical panels and sums the force contribution from each panel. The net drag force is given by:

$F_{D}=\frac{1}{2}\rho_{w}\sum_{i\in\left\{ b,r,l \right\}} \sum_{j=1}^{N} C_{D}{v\left( r_{j} \right)}^{2}c\left( r_{j} \right)\hat{\boldsymbol{v}}\left( r_{j} \right)\delta r$. (19)

Here $\rho_{w}$is the water density, $C_{D}$ is the drag coefficient, $v\left( r_{j} \right)$ and $c\left( r_{j} \right)$represent the local speed and chord length of the $j^{\mathrm{th}}$ panel, and $\delta r$ is the panel width. We do not include contributions from rotational circulation and added mass because their force coefficients are not quantified in previous studies. Inclusion of these terms may lead to unnecessary over-fitting. In this study, there is no lift force because the flaps are not allowed to rotate along the y-axis, which implies the angle of attack is always 90º. To gain physical insight about the torque induced on the passive flaps, we can further calculate the generalized forces through Supplementary Equation 17.

**Forces from flap flexures**

We model the flap flexure as a torsional spring with viscous damping. The torques exerted by the hinges along flap y-axes are given by:

$\tau_{ar}=-K_{h}\alpha_{r}-D_{h}\dot{\alpha}_{r},$ (20)

$\tau_{\alpha l}=-K_{h}\alpha_{l}-D_{h}\dot{\alpha}_{l},$ (21)

where $K_{h}$ is the stiffness and $D_{h}$ is the viscoelastic damping. Both parameters are dependent on the flap hinge geometry.

In addition, the flap rotation is limited in one direction. As shown in Figure 1, the flexure support structure behaves as a mechanical stop that prevents the flap from rotating in one direction. Hence, in the simulations, we limit the range of motion of the right and the left flaps to $\alpha_{r}<6^{\circ}$ and $\alpha_{l}>-6^{\circ}$, respectively. Once the rotation angles exceed these limits, we explicitly modify the rotational speed by the equations:

$\begin{matrix} \dot{\alpha}_{r}=\min\left( \dot{\alpha}_{r},0 \right), \\ \dot{\alpha_{l}}=\max\left( \dot{\alpha}_{l},0 \right). \end{matrix}$ (22)

While these equations enforce the geometric constraints, they do not model the impact due to the collision of the hinge support structures.

**Supplementary Note 2: Motion tracking of flapping experiments in water**

We conduct single leg flapping experiments in water to quantify the flapping kinematics and determine appropriate design parameters such as flexure stiffness and flap area. A single leg setup is constructed (Supplementary Fig. 2a) whose transmission ratio and flexural stiffness are identical to that of the robot (Fig. 1a). A Phantom V 7.10 high speed camera is placed above the flapping experiment and it records 200 images per flapping period. We use the commercial package Proanalyst to track the positions of the right flap tip, the leg body, and the leg root for every image (Supplementary Fig. 2b). Given these three positions, we can compute the kinematic parameters through the following equations:

$\begin{matrix} \psi=\tan^{-1} \frac{lr_{x}}{-lr_{y}}, \\ \begin{matrix} \alpha_{r}=-\cos^{-1} dot\left( \hat{\boldsymbol{fl}},\hat{\boldsymbol{lr}} \right), \\ x=\left| \boldsymbol{lr} \right|\sin\psi, \end{matrix} \\ y=\left| \boldsymbol{lr} \right|\cos\psi. \end{matrix}$ (23)

Here $\boldsymbol{lr}$ and $\boldsymbol{fl}$ are displacement vectors from the leg root to the leg and from the leg to the flap tip, respectively. The kinematics of the left flap is not measured because it is visually occluded by the leg transmission (Supplementary Fig. 2b).

**Supplementary Note 3: Comparison of experiments and simulations**

Our experiments only quantify flapping kinematics but do not measure the corresponding drag profiles. We use the quasi-steady model to estimate the drag force generated by the leg and the passive flaps. Using this model, we aim to design an input leg motion that improves time averaged thrust force under the constraint of the actuator’s maximum output force.

We validate the quasi-steady model before using it for the design of leg motion. We compare the simulated passive kinematics of the flap with the measured values (Supplementary Fig. 2d, e). Using the setup shown in Supplementary Figure 2a, we drive the actuator using sinusoidal inputs with voltage amplitudes from 100 to 200V, and with frequencies from 1 Hz to 7 Hz. We record high speed videos and extract the leg and flap kinematics for each experiment. The extracted leg motion is used as inputs to our simulations and we solve for the flap’s passive pitching motion and the net force. The mean error between the measured and the simulated pitching amplitude is 4°. We further compare the phase shift between the leg motion and the passive flap rotation (Supplementary Fig. 2e). The average phase shift difference between the experiments and the simulations is 0.06 period. Overall, the experiments and the simulations show similar trend, despite noticeable error between the measured and simulated phase shift.

Having validated the model, we further use the simulation to estimate force and improve the input leg motion. The blue curve in Supplementary Figure 2f shows the drag force profile for a 200 V, 5 Hz driving signal. The drag force during the downstroke and the upstroke approximately cancels each other, leading to a small net thrust of 0.015mN. This simulation shows that symmetric driving functions are ineffective for swimming. By designing an asymmetric driving input shown in Figure 4c, the model predicted net thrust force increases substantially. The red curve in Supplementary Figure 2f shows the drag force profile for an asymmetric input shown in Figure 4c. The net thrust increases to 0.13 mN – eight times improvement compared to the symmetric driving input. This input signal is used for the robot swimming experiments shown in Figure 5a.

**Supplementary References**

1. Spong, M. W., Hutchinson, S. & Vidyasagar M. Dynamics. *Robot Modeling and Control,* Wiley, **3**, 239–285 (2006).
