# Supplementary material for: Controllable water surface to underwater transition through electrowetting in a hybrid terrestrial-aquatic microrobot
Source: Nat Commun. 2018 Jun 27;9:2495. doi: 10.1038/s41467-018-04855-9 (PMC6021446; doi:10.1038/s41467-018-04855-9)
Supplement: Supplementary file 10 — Description of Additional Supplementary Files [file 41467_2018_4855_MOESM10_ESM.pdf]

Description of Additional Supplementary Files

File Name: Supplementary Movie 1
Description: Demonstration of hybrid terrestrial-aquatic locomotion and transitions. This movie shows end-to-end demonstrations in which the robot starts walking on land, transitions onto the water surface, swims on water, controllably sinks into water at desired locations, walks underwater, and finally climbs an incline to transition back onto land. Part 1 shows the top and side views. Two drops of blue dye are added to the water to enhance the side view movie. Part 2 shows a similar demonstration in clear water.

File Name: Supplementary Movie 2
Description: Transition from land to the surface of water. Perspective and top view videos show the robot walking down an incline and transitioning onto the surface of water. The robot changes to a swimming gait once it reaches the water surface.

File Name: Supplementary Movie 3
Description: Demonstration of robot locomotion on the water surface. Part 1 shows the robot swims across a 45 cm long tank in 15 seconds. Part 2 shows the robot turning left and then right on the water surface. Part 3 shows a high-speed video of the robot swimming.

File Name: Supplementary Movie 4
Description: Controllable sinking. Part 1 shows the robot cannot sink into water by violently paddling its legs to agitate the water surface. Part 2 shows the front and perspective views of the robot sinking into water when its EWPs are actuated. Part 3 shows the spontaneous wetting process of water on a copper sheet coated with 15 μm Parylene C.

File Name: Supplementary Movie 5
Description: Underwater locomotion. When fully submerged, the robot uses terrestrial walking gaits to demonstrate forward motion and turning.

File Name: Supplementary Movie 6
Description: Underwater to land transition. Part 1 shows the top and side views of the robot crawling up a 6° incline covered by 1200 grit sandpaper. Part 2 shows the side view of the robot crawling up a 3° acrylic incline.

File Name: Supplementary Movie 7
Description: Adverse surface tension effects during underwater to land transition. Without design modifications, the robot cannot break the water surface and walk back onto land. This video shows the robot getting stuck on the incline as its front part pushes out from the water surface.
