# Supplementary figures and images for: Controllable water surface to underwater transition through electrowetting in a hybrid terrestrial-aquatic microrobot
Source: Nat Commun. 2018 Jun 27;9:2495. doi: 10.1038/s41467-018-04855-9 (PMC6021446; doi:10.1038/s41467-018-04855-9)

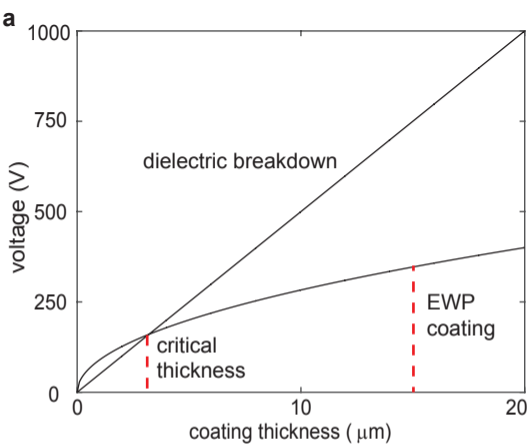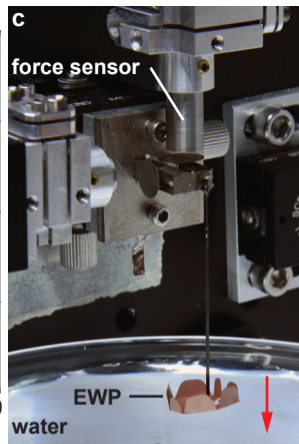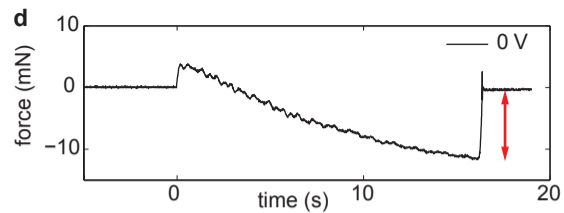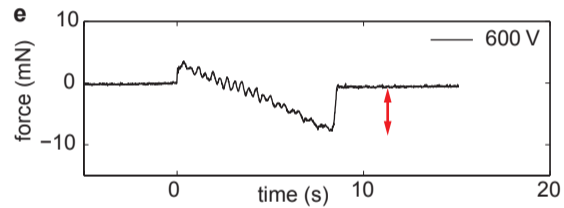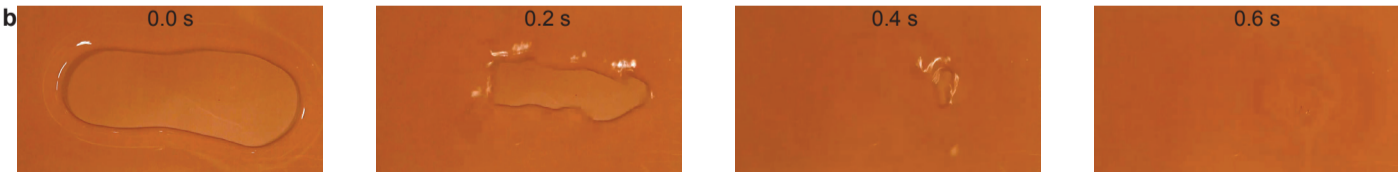

Supplement: Supplementary file 11 — Supplementary Figure 1 [file 41467_2018_4855_MOESM11_ESM.pdf]

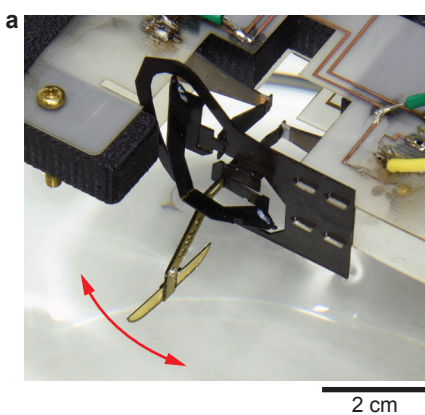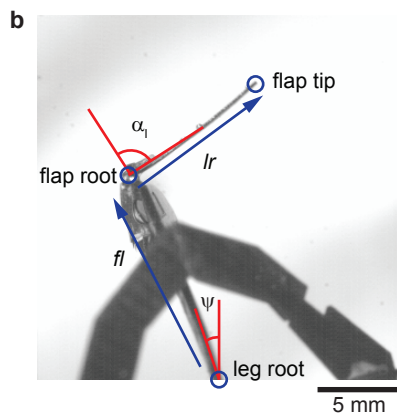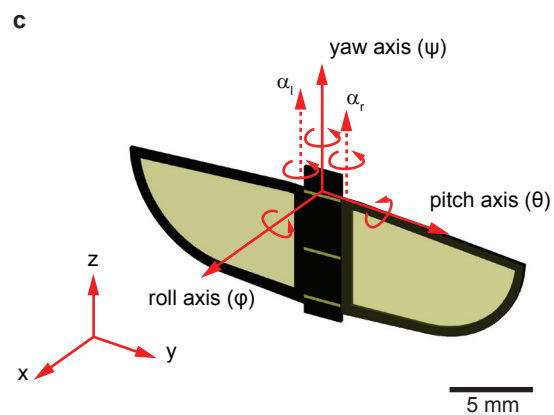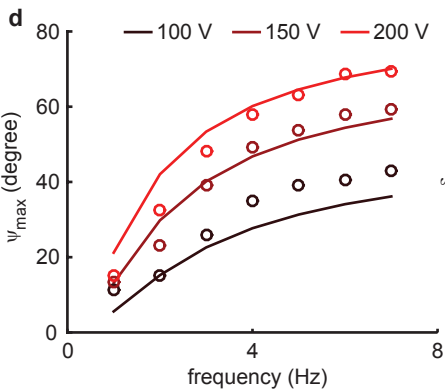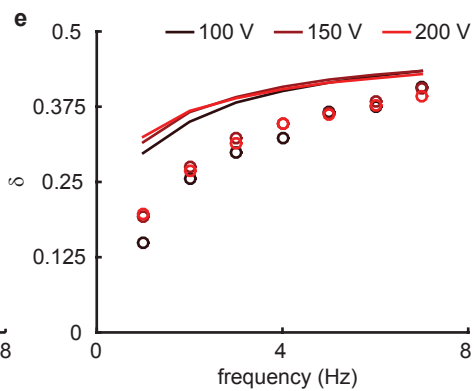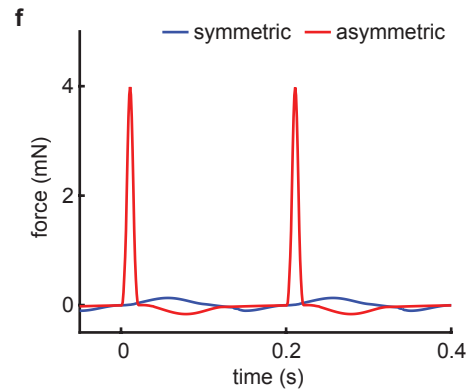

Supplement: Supplementary file 12 — Supplementary Figure 2 [file 41467_2018_4855_MOESM12_ESM.pdf]

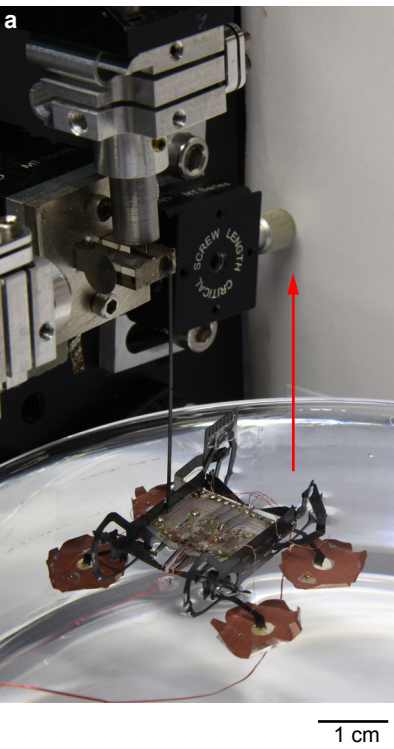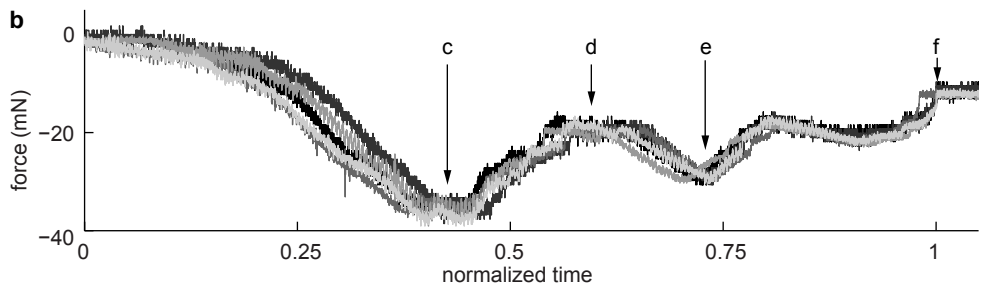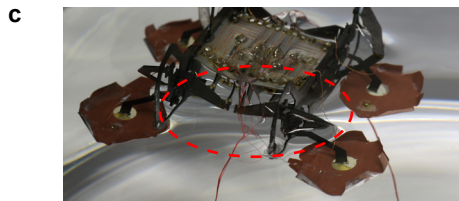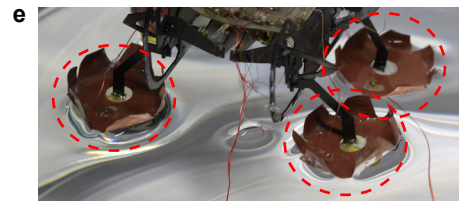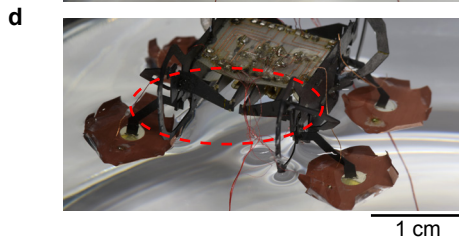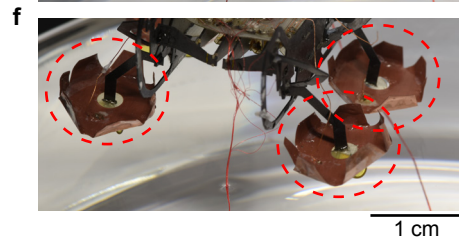

Supplement: Supplementary file 13 — Supplementary Figure 3 [file 41467_2018_4855_MOESM13_ESM.pdf]

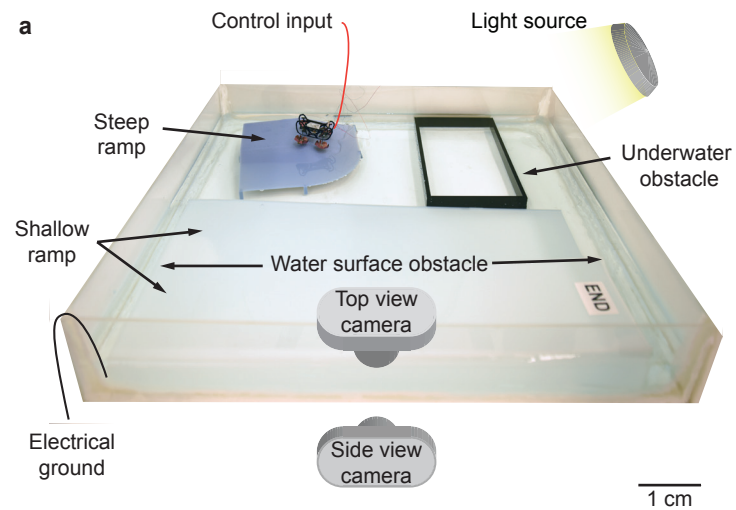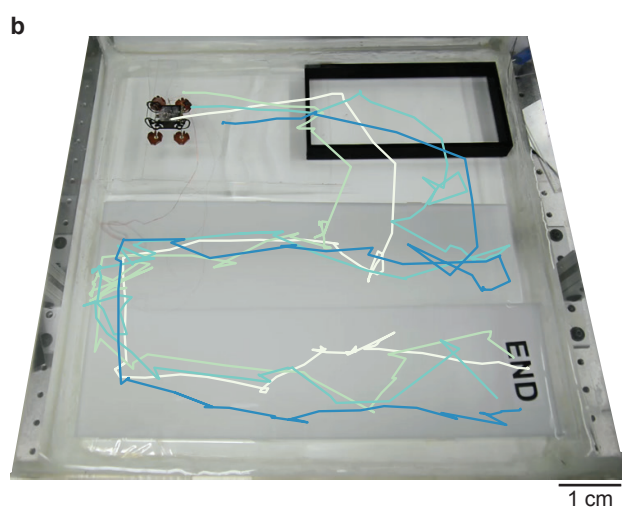

Supplement: Supplementary file 14 — Supplementary Figure 4 [file 41467_2018_4855_MOESM14_ESM.pdf]
